# Drought Stress in Roses: A Comprehensive Review of Morphophysiological, Biochemical, and Molecular Responses

**DOI:** 10.3390/ijms26094272

**Published:** 2025-04-30

**Authors:** Hmmam Zarif, Chunguo Fan, Guozhen Yuan, Rui Zhou, Yufei Chang, Jingjing Sun, Jun Lu, Jinyi Liu, Changquan Wang

**Affiliations:** 1 College of Horticulture, Nanjing Agricultural University, Nanjing 210095, China; hmmam.zareef@agr.menofia.edu.eg (H.Z.); 2021204054@stu.njau.edu.cn (C.F.); 2022204020@stu.njau.edu.cn (G.Y.); zr202020@163.com (R.Z.); 2024204082@stu.njau.edu.cn (Y.C.); t2021068@njau.edu.cn (J.S.); lujun123@njau.edu.cn (J.L.); jyl@njau.edu.cn (J.L.); 2 Key Laboratory of Landscaping, Ministry of Agriculture and Rural Affairs, Nanjing 210095, China; 3 Key Laboratory of State Forestry and Grassland Administration on Biology of Ornamental Plants in East China, Nanjing 210095, China; 4 Horticulture Department, Faculty of Agriculture, Menoufia University, Shebin El Koum 32514, Egypt

**Keywords:** climate change, water scarcity, roses, molecular responses, essential oil, post-harvest

## Abstract

Climate change poses significant threats to agriculture globally, particularly in arid and semi-arid regions where drought stress (DS) is most severe, disrupting ecosystems and constraining progress in agriculture and horticulture. Roses, valued for their aesthetic appeal, are highly susceptible to abiotic stresses, especially DS, which markedly reduces flower quantity and quality. Under DS conditions, roses exhibit diverse morphological, physiological, biochemical, and molecular adaptations that vary across species. This review examines the effects of DS on rose growth, yield, and physiological traits, including gas exchange, photosynthesis, phytohormone dynamics, and water and nutrient relationships, alongside their biochemical and molecular responses. Furthermore, DS impacts the biosynthesis of secondary metabolites, notably reducing the yield and quality of essential oils in roses, which are critical for their commercial value in perfumery and aromatherapy. Additionally, the impact of DS on rose flower quality and post-harvest longevity is assessed. By elucidating these diverse responses, this review provides a framework for understanding DS effects on roses and offers insights to develop strategies for mitigating its adverse impacts.

## 1. Introduction

**Figure 1:**
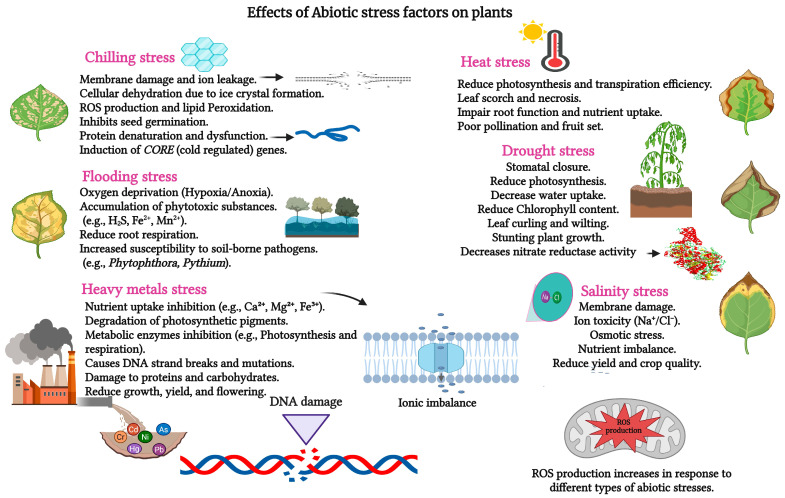
Different kinds of abiotic stresses and their effects on plants. Macronutrients: Ca^2+^—Calcium. Micronutrients: Fe^2+^—Ferrous Iron, Fe^3+^—Ferric Iron, Mn^2+^—Manganese, Ni—Nickel, Na^+^—Sodium, Cl^−^—Chloride. Toxic Elements: Pb—Lead, As—Arsenic, Cd—Cadmium, Cr—Chromium, Hg—Mercury. Signaling Compound: H_2_S—Hydrogen Sulfide.

Plants thrive in dynamic environments that often impose challenging conditions on their growth and development. Environmental stressors, classified as biotic or abiotic, significantly impact plant life, with abiotic stressors such as drought and salt stress (Figure 1) impairing growth, development, and productivity [1,2]. DS, a critical stressor in horticulture and floriculture, reduces crop yield, limits growth, and disrupts the synthesis of secondary metabolites—an escalating concern amid evolving global climate patterns [3,4,5,6,7]. The Intergovernmental Panel on Climate Change (IPCC) projects that this trend will continue, with average temperatures expected to rise by 1.8–4.0 °C by 2100, exacerbating drought prevalence in many regions and posing a significant threat to agricultural productivity and efficiency [8]. This scenario presents a major challenge for the scientific community aiming to develop sustainable strategies to mitigate DS impacts and ensure global food and horticultural security.

Roses (*Rosa* spp. L.), a key genus in the Rosaceae family, encompass nearly 200 species and over 30,000 modern cultivars distributed across diverse climatic zones globally [9,10,11,12]. Its value is due to its economic, cultural, and symbolic significance; roses dominate floriculture, with cut roses comprising 32% of the global cut flower market [13]. The global rose market, valued at USD 525.94 million in 2023, is projected to reach USD 1092.77 million by 2032, with a compound annual growth rate (CAGR) of 8.7% from 2024 to 2032 [14]. Europe held the largest market share in 2023, led by the Netherlands, the top exporter with a USD 939 million market share, followed by Ecuador (USD 850 million) and Kenya (USD 581 million), where rose cultivation supports millions of livelihoods [15]. Their adaptability to varied environments and climates makes them a preferred choice for cultivation in gardens, parks, public spaces, and floriculture [16,17]. For centuries, their diverse colors, shapes, and fragrances have made them a favored flower, widely used as cut flowers and perfume ingredients, and a valuable resource in the medical, cosmetic, and food industries, rendering them a profitable agricultural crop in many countries [17,18]. However, their wide geographical distribution and extensive cultivation increase their vulnerability to adverse environmental conditions, particularly DS, significant abiotic stress in horticulture and floriculture that threatens global rose production by impairing growth, yield, and the biosynthesis of economically critical compounds, thereby affecting both quantity and quality [3,4,19,20]. Water scarcity, caused by insufficient rainfall or uneven distribution of moisture, accounts for about 70% of global crop yield and productivity losses, posing a serious threat to agriculture and floriculture [21,22,23].

For roses, a valuable ornamental crop, developing effective DS management strategies and predicting plant responses requires a comprehensive understanding of their multifaceted responses to DS, encompassing morphological, physiological, biochemical, and molecular dimensions, as examined in this review. Insights into roses’ adaptive and survival mechanisms in arid environments will enhance our understanding of plant responses to abiotic stress, facilitating the development of targeted strategies to mitigate DS impacts.

## 2. Plant DS: Causes and Classification

### 2.1. Causes of Water Deficiency in Plants

DS arises when the soil water available to plants diminishes over time due to insufficient soil moisture, driven by climate change-related factors such as altered precipitation patterns, elevated atmospheric carbon dioxide levels, and rising air temperatures [24,25,26]. High-intensity light and dry tropical winds further exacerbate DS by accelerating soil water evaporation and dehydration [27]. Notably, plants can experience DS even when soil moisture is adequate, as environmental conditions like salinity, low soil temperatures, or waterlogging impair root water absorption, leading to a condition known as physiological drought or pseudo-drought [24,28].

### 2.2. Classification of DS

DS can be categorized into three severity levels based on relative water content (RWC), a reliable indicator of drought intensity: mild (RWC 60–70%), moderate (RWC 40–60%), and severe (RWC 0–40%). Severe DS significantly impairs plant function, reducing photosynthetic activity, inhibiting growth and development, and, with prolonged exposure, ultimately leading to plant death [29].

## 3. Effect of DS on the Morphology, Physiology, and Biochemistry of Roses

**Figure 2:**
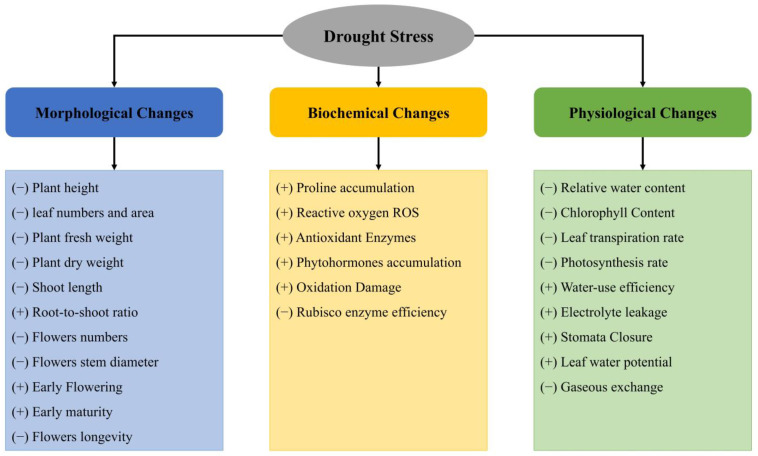
Effects of DS on the morphology, physiology, and biochemistry of roses. (+) Induce; (−) Reduction. Partially adapted from Seleiman et al. [32].

DS exerts complex effects on plants, impacting a wide range of morphological, physiological, biochemical, and molecular characteristics and processes, thereby impairing plant functions at any growth stage affected by DS [30,31]. Figure 2 illustrates the diverse effects of DS on rose plants.

### 3.1. Drought-Morphological Attributes in Roses

A lack of water induces significant morphological changes in plants, including reduced fresh and dry weight, decreased leaf area, increased leaf thickness, leaf curling, wilting, accelerated senescence, stomatal closure, and uneven leaf surface stratification [33,34,35,36]. Numerous studies have shown that increasing DS severity leads to a corresponding decline in rose growth parameters and biomass. For example, the Damask rose (*R. damascena* Mill.), a member of the Rosaceae family originating from Damascus and valued for its aromatic properties in the global cosmetics and pharmaceutical industries, exhibited significant reductions in leaf number, leaf area, and fresh and dry biomass under DS conditions [37,38]. Similarly, DS caused a notable decrease in fresh and dry leaf weight and biomass percentage in the Damask rose cultivars Maragheh and Kashan [39]. In another study, *R. damascena* var. Dieck trigintipetala was subjected to three water availability levels over 90 days: optimal conditions at full field capacity (FC), mild DS at 50% FC, and severe DS at 25% FC. The plants displayed reduced overall growth, with significant decreases in total fresh and dry weight, greater under severe DS than mild DS, although leaf number remained unaffected [40]. 

Damask roses subjected to varying levels of water deprivation (70%, 40%, and 10% water availability) exhibited significant declines in growth and flowering characteristics from June to October. DS increased the root-to-shoot ratio throughout the experiment while negatively impacting other growth parameters, including a reduction in main stem length (up to 49.7%), canopy diameter, number of leaves (up to 54%), individual leaf area (up to 64%), total plant leaf area (up to 83%), and overall shoot dry weight, severely limiting plant growth. DS also adversely affected all measured floral traits, such as time to flowering, number of flowers, flower diameter, individual flower dry weight, number of petals, receptacle diameter, and individual petal dry weight. Furthermore, reduced soil water availability accelerated the growth cycle, leading to earlier flowering by up to 7.4 days [41]. In addition, Shi et al. [42] determined that water scarcity contributed to the lower total fresh weight of *R. hybrida* cv. Charming Black plants in comparison with those exposed to natural irrigation. Williams et al. [43] further demonstrated that even a partial water supply (60–75% of requirements) significantly decreases the fresh-to-dry weight ratio in miniature rose cultivars. In their study, the authors confirmed that water restriction decreased the amount of water in plant tissues, thus reducing the fresh-to-dry weight ratio. A consistent association was established between water deficiency (WD) and a significant reduction in shoot length, shoot weight, and leaf area at different growth stages in the cut rose cultivar Charming Black [42]. *R. hybrida* cv. Club Nika was exposed to three irrigation schemes (100%, 50%, and 25% of the irrigation requirement), aiming to determine the impact of dehydration on their morphological and qualitative traits. Water-deficient plants showed significantly decreased fresh and dry flowering stem weight than well-irrigated seedlings. The decreases were 19% and 36% at 75% and 50% irrigation levels, respectively. The bud sprouting occurred faster (about 6.83 days) in well-irrigated plants than under stress conditions [44]. As a result of dehydration conditions, the fresh and dry weight of rose seedlings decreased. It is worth noting that the seedlings subjected to extreme water deprivation stress had significantly lower dry weights than those exposed to normal and mild drought stress treatments [45]. 

In a parallel investigation on roses conducted by Farahani et al. [46], extreme WS led to a 40% decrease in leaf numbers and total leaf area. Conversely, mild stress did not affect leaf quantity but caused a 20% reduction in the dimensions of each leaflet. Katsoulas et al. [47] studied how irrigation frequency affects the dry and fresh weights of cut roses. They proved that high frequencies increased cut flower buds’ dry and fresh weights by 33% compared to low frequencies. Nevertheless, no significant differences in the number of flower stalks were recorded across three distinct irrigation levels [48]. Additionally, Raviv et al. [49] stated that DS induces stomatal closure, decreasing plant turgor and inhibiting expansion growth.

For instance, a decrease in soil moisture content in rose plants reduces shoot length. This finding is consistent with research conducted by Sotelo-Cuitiva et al. [50] on rose seedlings. Water scarcity negatively impacts the quality and amount of roses produced, causing a 20% drop in flower production compared to plants that receive full irrigation, as shown by Farahani et al. [51]; additionally, all water scarcity treatments reduce the number of flower buds. Similarly, it was noted that under fully irrigated conditions, the yield of *R. alba* L. fluctuated between 3930 and 7700 kg/ha. In contrast, in non-irrigated conditions, it exhibited the same range of 3930 to 7700 kg/ha [52]. Finally, fresh and dried flower weights, quantity, and width significantly declined in water-deficient rose plants relative to adequately irrigated ones [38].

The root system is crucial for plant growth, forming a root–soil interface that enables water and nutrient uptake, particularly under abiotic stresses like drought. Roots are the first to detect soil drying, triggering chemical, hydraulic, and molecular signals that modify plant morphology, phenology, and physiology [53,54,55]. Soil drying exacerbates soil impedance, alters texture, reduces water-holding capacity, and restricts root development [56], often triggering adaptive responses such as an increased root-to-shoot ratio to prioritize water acquisition [57]. In roses, water stress (WS) responses vary significantly across species and cultivars. For example, *R. damascena* and *R. odorata* exhibited higher root-to-shoot dry weight ratios under water deprivation, driven by a significant reduction in shoot growth compared to roots [58,59]. Similarly, *R. multiflora* seedlings under extreme drought (0–10% soil moisture) allocated 1.4 times more biomass to roots than under high water conditions, reflecting a drought tolerance strategy [60]. Conversely, DS reduced the root-to-shoot ratio in some rose cultivars. Al-Yasi et al. and Bolla et al. [40,61] reported significant dry-weight ratios and reduced stem biomass declines, while Cai et al. [62] observed impaired root growth in Belinda’s Dream and Marie Pavie’s garden roses. In contrast, *R. hybrida* cultivars showed increased root dry weight under WS compared to controls, highlighting genotypic variability in WS responses [63]. In *R. hybrid* Cv. Rouge Meiland, WS induced by polyethylene glycol (PEG 6000) stimulated root elongation but significantly reduced root biomass. Interestingly, the total root volume and surface area showed no significant differences across treatments [64]. These contrasting findings underscore the need for further research into the genetic and environmental factors that lead to morphological differences in rose root adaptations under DS.

Water shortage adaptive morphological changes in plants, often increasing the root-to-shoot ratio to enhance root system growth while limiting aerial parts in response to reduced water availability [57]. In *R. damascena* Mill., researchers observed an increase in the dry weight root-to-shoot ratio under DS [54], though Al-Yasi et al. and Bolla et al. [40,61] reported a significant decrease in this ratio in roses. DS also reduced the number of rose stems compared to well-irrigated plants. However, it did not affect the number of high-quality stems or the average length and weight of flower stems, indicating that while DS impacts overall stem production, it does not compromise individual flower stem quality.

Leaf morphology is notably affected by DS, as demonstrated by Jia et al. [65], where *R. chinensis* Jacq. cv. Old Blush plants subjected to DS for 15, 30, 45, 60, 75, and 90 days, followed by 7 days of rehydration, showed progressive changes: leaves remained intact after 15 days, developed slight wrinkling after 30 days, and exhibited severe wrinkling and wilting by 45 to 90 days, but fully recovered, turning green and thin, after rehydration. Rose species exhibit varying DS tolerance; for instance, cyclic DS testing on four rose rootstocks (*R. fortuniana*, *R. multiflora*, *R. odorata*, and *R. hybrida* cv. Dr. Huey) revealed that *R. fortuniana* displayed superior vegetative growth parameters and greater leaf area, while *R. odorata* experienced severe growth reduction, with *R. multiflora* and cv. Dr. Huey showed intermediate responses, *R. fortuniana* being the most drought-tolerant [59]. Blum et al. [66] identified mechanisms reducing leaf area under DS, including decreased cell proliferation and division, leaf curling, and necrosis of apical regions and entire leaves, contributing to the typical DS-induced reduction in leaf area. Additionally, a study on DS tolerance in *R. rugosa* genotypes (wild type [W] and five lines) showed that the W exhibited the highest tolerance due to structural adaptations such as sunken stomata, robust tissue, and low stomatal density, which minimize water loss and enhance DS resistance [67].

Collectively, the results highlight the negative impact of WD on the morphological traits of rose plants. WD affects bud development, growth, leaf structure, and overall growth, reducing yield and poor crop quality.

### 3.2. Physiological and Biochemical Responses

#### 3.2.1. Gaseous Exchange

A significant consequence of dehydration in crops is the alteration of gas exchange processes, critical for photosynthesis and respiration, primarily through enhanced stomatal closure and reduced gas exchange due to limited water availability [68]. Key indices for assessing these changes include stomatal conductance (Gs), transpiration rate (E), intracellular CO_2_ content (Ci), and net photosynthesis (Pn) [69]. In *R. damascena* Mill., mild DS 50% FC increased Pn and Gs by 31% and 19%, respectively, compared to optimal irrigation, while severe DS 25% FC reduced them by 55% and 36%, respectively [40]. Similarly, Li et al. [45] reported significant declines in Pn, E, and Gs under DS in *R. chinensis* cv. Mutabilis. Miniature rose cultivars under DS exhibited 25% lower Pn and 63% lower Gs than well-irrigated plants. However, Gs and Pn increased during recovery but remained 35% and 8% lower than the control group [43]. Moreover, Sotelo-Cuitiva et al. [50] indicated that rose seedlings under DS had lower Gs, with leaf photosynthetic indices (Pn, Gs, E) significantly affected, though Ci remained constant; higher DS levels also reduced Pn by 27% compared to adequately irrigated plants [43]. Moreover, higher water-stressed plants showed a 27% decrease in Pn compared to adequately irrigated plants [44]. Additionally, a study on six *R. rugosa* genotypes under DS, induced by varying levels of PEG-6000, showed reductions in E, Pn, and Gs, with a 5% PEG treatment causing a 29.42% decrease in E; the wild-type genotype displayed superior drought tolerance, as evidenced by higher Pn and Gs values [67].

#### 3.2.2. Photosynthesis Parameters: Chlorophyll Content and Photosynthesis

**Figure 3:**
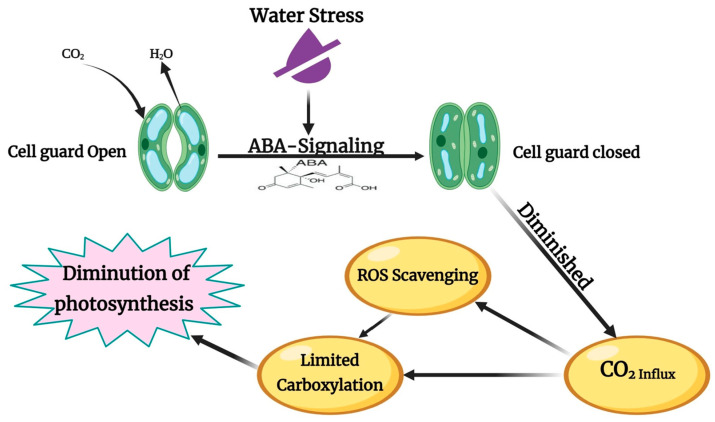
Effect of drought on the photosynthesis process, modified from Farooq et al. [74]. ABA—Abscisic acid. CO_2_—carbon dioxide. H_2_O—water.

Photosynthesis is a fundamental metabolic process that profoundly affects plant growth and production. Drought can significantly affect this process by impeding plants’ typical photosynthesis rate and gas exchange [70]. Limited water availability causes stomatal closure, which restricts the delivery of carbon dioxide (CO_2_) to the leaves—an essential component for photosynthesis—and promotes the production of reactive oxygen species (ROS) [71], as illustrated in Figure 3. In addition, water deficiency (WD) increases leaf temperature, inhibiting the synthesis of photosynthetic pigments [72]. DS also diminishes the activity of key photosynthetic enzymes, such as Rubisco, slowing down photosynthesis and damaging its machinery [73]. According to Mishra et al. and Sher et al. [35,68], plant pigments, particularly chlorophyll, are vital for photosynthesis as they absorb light and generate reducing power. DS hampers the ability of mesophyll cells to utilize available CO_2_, leading to a reduction in chlorophyll content, as reported by Sarwar et al. [69]. A study on Damask roses demonstrated that increasing DS decreased total chlorophyll (T Chl), chlorophyll A, and chlorophyll B concentrations. Among these, chlorophyll B was most affected, exhibiting a 37% reduction under mild water stress (MWS) and a 54% reduction under severe water stress (SWS). Conversely, the chlorophyll A/B ratio increased by 29% under MWS and 46% under SWS conditions [37].

The findings align with Seyed Hajizadeh et al. [39], who reported that as the PEG concentration increased, Damask rose genotypes of the cultivars Maragheh and Kashan’s chlorophyll content decreased by up to 30% and 41%, respectively, suggesting that some rose genotypes may be more resistant to WS than others. In contrast, Adamipour et al. [75] found elevated chlorophyll content in *R. canina* L. and *R. damascena* Mill. under DS compared to well-watered conditions, suggesting adaptive traits like *R. canina’s* genetic capacity to retain green leaves during stress. However, studies by Dolatkhahi et al. and Li et al. [44,76] observed no significant impact of water deprivation on photosynthetic pigments in rose plants. On the other hand, *R. hybrida* cv. Charming Black [77], *R. rubiginosa* [78], and *R. damascena* cv. Cachan 93 [41] exhibited reduced ph-osynthetic pigments under water-deficit conditions, which was attributed to impaired photosynthetic mechanisms.

#### 3.2.3. Water-Relations

WD significantly impacts plant-water interactions, affecting key parameters such as RWC, water use efficiency (WUE), transpiration rate (Tr), leaf water potential (ψ), stomatal resistance, membrane integrity index, and leaf temperature, all of which are critical for plant growth [74,79]. RWC is an essential indicator of plant hydration, reflecting the metabolic activity in tissues and providing a valuable measure for assessing plant resilience to (DS) [74]. Furthermore, Farooq et al. [31] suggest that leaf water potential (LWP) is a useful tool for evaluating the extent of soil WS experienced by plants, offering critical insights into the plant-water relationship. According to Noctor et al. [80], RWC is an early response to DS, followed by a decline in leaf water potential (LWP) and stomatal closure. DS reduces RWC and LWP in Damask roses, intensifying effects under severe conditions [37,40]. Under severe water stress (SWS), leaf water potential decreased by 72% compared to the control, while mild water stress (MWS) resulted in a 46% reduction; additionally, leaf water content (LWC) decreased significantly by 17% under SWS [40]. Adamipour et al. and Alavi et al. [75,81] observed consistent DS impacts in *R. hybrida* cv. Red, *R. canina*, and *R. damascena*, while De Dauw et al. [82] reported comparable results in tetraploid and diploid roses under water scarcity. Li et al. [76] found that the LWC of 2-month-old rose seedlings decreased significantly under MWS and SWS, reaching 41% and 15% of the control, respectively. A 90-day water deficit in *R. chinensis* cv. Old Blush led to the lowest recorded values for LWC and soil water content (SWC) [65], a trend also observed by Sotelo-Cuitiva et al. [50] in Charlotte roses grafted onto Natal Briar under DS. Additionally, rose cultivars Poul Happy Charming Parade and Polabian Bianca Parade exhibited higher WUE under DS compared to unstressed plants [43], while excessive irrigation led to lower WUE values [45,49,61,83]. This is consistent with findings by Shangguan et al. and Kapoor et al. [84,85], who noted that drought-stressed plants often have elevated WUE. However, Aalam et al. [41] reported that soil water deficits don’t consistently affect photosynthetic or shoot WUE in roses, highlighting variability in these responses.

#### 3.2.4. Nutrient Relations

In addition to its detrimental effects on plant growth and productivity, DS impairs nutrient uptake by limiting water availability. Water shortage reduces nutrient absorption by roots and their subsequent translocation to shoots [86,87,88], primarily due to decreased root growth, reduced nutrient flow per unit root length, and lower root biomass under low soil moisture conditions [89]. DS negatively impacts the uptake of essential minerals, including nitrogen (N), phosphorus (P), calcium (Ca), silicate, and magnesium (Mg), often resulting in stunted growth and development in stressed plants [90]. A recent study on roses revealed that irrigation levels influence nutrient accumulation in leaves, with nutrients such as potassium (K^+^), P, Mg, and zinc (Zn) showing reduced accumulation in shoots under a subsurface drip irrigation (SDI) system operating at 50% FC [81]. Similarly, in *R. damascena* Mill., DS induced by PEG decreased leaf K^+^ and P contents by 56% and 52% in the Maragheh cultivar and by 47% and 52% in the Kashan cultivar, respectively, while iron (Fe) content dropped by up to 49% in Maragheh and 51% in Kashan with increasing PEG levels [39]. However, previous research on drought-stressed rose leaves reported variable accumulation of inorganic elements, with K^+^ levels increasing while Ca and chloride (Cl^−^) either decreased or remained unchanged [40]. Elevated K^+^ concentration plays a critical role in drought tolerance by regulating osmotic pressure, modulating membrane potential, facilitating CO_2_ fixation, supporting the translocation of photosynthetic products to sink organs, and reducing electron transfer to O_2_, thereby lowering reactive oxygen species (ROS) levels and promoting sustainable growth and yield under arid conditions [91,92]. Furthermore, Luo et al. [93] found that adequate water availability significantly reduced N and P levels in the leaves of two *R. roxburghii* cultivars (Gui 2 and Gui 7), while DS had no significant effect on leaf K^+^ levels.

#### 3.2.5. Phytohormone Regulation

Plant hormones, or phytohormones, are essential regulators of plant growth, development, and responses to abiotic stresses, particularly drought and salinity [94,95,96]. They mitigate the adverse effects of DS through physiological and developmental adaptations, such as maintaining osmotic balance, inducing stomatal closure, and regulating root growth to enhance water uptake [95,97,98,99]. The mechanisms of action vary by hormone [100], with abscisic acid (ABA), gibberellins (GAs), ethylene (ET), jasmonates (JA), and salicylic acid (SA) enhancing DS tolerance by altering molecular processes through cell signaling pathways [101]. For instance, Li et al. [45] found that ABA, a primary drought-responsive hormone, increases in rose leaves and roots during DS adaptation, underscoring its role in stress response.

Additionally, auxins promote DS tolerance by stimulating lateral root formation to enhance water absorption and by upregulating stress-responsive genes, which increase ABA synthesis and modulate antioxidant enzyme activity, thereby reducing water loss [102,103]. Notably, higher ABA levels and lower GA concentrations are associated with improved plant resistance to DS [104]. Table 1 summarizes the roles of phytohormones in rose plants under DS conditions. Moreover, phytohormones regulate plant responses to DS by modulating transcription factors (TFs), a key step in cell signaling pathways. In *R. hybrida*, the DS-tolerance gene *RhMED15a* is upregulated by ABA treatment, enhancing its expression, whereas methyl jasmonate (MeJA) downregulates *RhMED15a*, suggesting that JA may reduce the gene’s sensitivity to DS [105]. Furthermore, changes in JA signalling pathways under DS indicate a broader role for JA in plant responses to drought stress [105].

#### 3.2.6. Oxidative Stress: Production of Reactive Oxygen Species and Adaptive Responses

Under abiotic stresses like DS, plants accumulate ROS, including hydrogen peroxide (H_2_O_2_), hydroxyl radicals (OH·), superoxide radicals (O_2_^−^), and singlet oxygen (^1^O_2_), which disrupt normal physiological functions [109,110,111]. At low concentrations, ROS serve as signaling molecules, activating stress-responsive pathways and enabling intercellular communication [112]. The interplay between ROS-producing and scavenging enzymes, along with the antioxidant system, maintains cellular redox homeostasis by regulating intracellular ROS concentrations in response to environmental stresses [112]. However, elevated ROS levels are phytotoxic, impairing plant growth and development by disrupting essential biological and physiological processes [113,114,115,116]. Excessive ROS cause oxidative damage through mechanisms such as lipid peroxidation, malondialdehyde (MDA) production, and cell death, targeting proteins, DNA, RNA, cellular membranes, and other biomolecules [109,117,118,119]. DS exacerbates this damage by increasing ROS production, leading to oxidative stress and amplifying the detrimental effects on plant cells [120]. ROS thus play a dual role as byproducts of oxygen metabolism, regulating cellular redox status and activating stress responses while contributing to oxidative damage under adverse conditions, a balance critical for plant survival [112].

Oxidative stress can be assessed by analyzing different substances, such as malondialdehyde (MDA), a byproduct of lipid peroxidation, hydrogen peroxide (H_2_O_2_) and electrolyte leakage (EL) [117,121,122]. In drought-stressed rose species, such as *R. damascena* and *R. canina*, elevated levels of MDA and H_2_O_2_ accumulate [75]. This is consistent with findings that *R. canina* exhibits higher H_2_O_2_ levels during DS [123]. Similarly, in *R. chinensis*, MDA concentrations peaked after 90 days of DS but decreased upon re-watering [65]. Several *R. rugosa* genotypes also showed increased MDA and reactive oxygen species (ROS) levels with increasing DS severity [67]. DS-induced rose petals exhibited higher H_2_O_2_ and MDA levels, which subsequently decreased after rehydration, mirroring observations in dehydrated and rehydrated cut roses [124]. Prolonged DS in cut roses led to O_2_^−^ accumulation in petals, potentially aiding in repairing cell membrane damage, with levels decreasing upon rehydration [125]. Under adverse conditions like DS, plants activate robust antioxidant defense mechanisms to mitigate ROS-induced cellular damage [126,127]. These mechanisms involve enzymatic antioxidants, such as ascorbate peroxidase (APX), guaiacol peroxidase (GPX), superoxide dismutase (SOD), dehydroascorbate reductase (DHAR), and glutathione reductase (GR), as well as non-enz-ymatic antioxidants, including glutathione (GSH) and ascorbate (AsA) [109,126,128,129]. These components work synergistically across subcellular compartments to neutralize ROS or regenerate antioxidants, often using energy from sunlight [109,130,131,132,133]. Adequate water supply maintains a balance between ROS production and neutralization, limiting the formation of harmful oxygen metabolites. However, DS disrupts this balance, causing a rapid increase in intracellular ROS levels. The plant’s stress response can be assessed by the presence and activity of antioxidant molecules and enzymes, as detailed in Table 2.

#### 3.2.7. Osmotic Adjustment (OA)

Adapting DS involves multiple morphological, physiological, and biochemical pathways [58,136]. Osmoregulation, a critical physiological mechanism, enables plants to mitigate the adverse effects of osmotic stress, such as DS and salinity, by synthesizing osmotically active compounds and ions, including organic acids, soluble sugars, proline, glycine betaine, phenolic compounds, calcium (Ca^2+^), potassium (K^+^), and chloride (Cl^−^) ions [137,138,139]. These osmoprotectants allow plants to sustain growth, photosynthesis, and cell division under water-scarce conditions by promoting intracellular water uptake and retention, maintaining cell turgor, protecting against dehydration-induced damage, and stabilizing subcellular structures like membranes and proteins [138,140,141,142,143]. Proline, in particular, plays a vital role in preventing protein degradation and stabilizing cellular structures [33,143]. For instance, *R. damascena* seedlings exposed to DS for 90 days exhibited elevated proline and soluble sugar levels compared to unstressed controls, with proline increasing by 9.9% and 34.6% and soluble carbohydrates by 22.8% and 33.6% under mild and severe DS, respectively [40]. Similarly, Adamipour et al. [123] reported a significant increase in proline levels in DS-stressed rose plants, rising from 14.5 mM under optimal conditions to 33.8 mM at 50% FC and 75.5 mM at 25% FC. Comparable trends were observed by Luo et al. [93] in *R. roxburghii* Tratt., though Dolatkhahi et al. [44] noted relatively stable proline levels in rose leaves under DS. Miniature roses (*R. hybrida*) subjected to repeated DS cycles demonstrated osmotic adaptation through a decreased osmotic potential, driven by the accumulation of solutes like proline, soluble carbohydrates, and K^+^ rather than Na^+^ [144]. Rose plants subjected to continuous deficit irrigation (SDI) exhibited a 23.71% increase in total soluble carbohydrates compared to those not receiving SDI. Additionally, leaves from SDI-treated plants displayed higher proline concentrations [81]. Additionally, in arid environments, *R. rubiginosa* displays elevated soluble carbohydrate levels and slower water loss compared to humid sites; however, under drought stress (DS), these carbohydrates primarily accumulate rather than being utilized for growth or secondary metabolism [78,106,145,146].

#### 3.2.8. Influence of DS on Rose Petal Essential Oil Yield and Composition

Roses are highly valued as cut flowers for their aesthetic appeal and significant economic importance, primarily due to the essential oils in their petals [147]. Rose oil, one of the most expensive essential oils globally, requires approximately 3000 kg of rose petals to produce 1 kg, reflecting its low yield [148]. The global rose oil market, valued at USD 1.6 billion in 2022, is projected to grow from USD 1.67 billion in 2023 to USD 2.5 billion by 2032, with an expected CAGR of 4.56% from 2024 to 2032 [149]. The distinctive fragrance of rose oil arises from volatile aromatic compounds, including monoterpenes like linalool, beta-citronellol, and geraniol, as well as stress-related compounds like benzaldehyde [150]. Citronellol and geraniol are critical for rose oil’s quality, making it a key ingredient in perfumery, cosmetics, and pharmaceuticals [151,152,153]. Thus, understanding the factors that influence the production and quality of this essential oil is crucial, especially for producers [154].

Soil WD negatively impacts essential oil yield, intensifying effects as WS increases [41]. However, Farahani et al. [51] reported a 20% increase in crucial oil concentration in rose flowers under water deficit compared to well-watered conditions. This enhancement under drought is supported by Yousefi’s study of 49 Iranian damask rose landraces [155], which showed higher flower yields and essential oil content in landraces from temperate, warm, and arid regions compared to those from cooler, semi-arid, or humid areas. The composition of essential oils, critical to their quality and economic value, is influenced by environmental stressors [156]. Water shortage increases compounds like linalool, citronellol, geraniol, eugenol, methyl eugenol, and dodecanoic acid, likely protecting plant cells from oxidative damage [50]. Kiymaz et al. [157] found that *R. damascena* essential oil maintained quality under reduced irrigation (I_1_._00_ to I_0_._50_), with gas chromatography–mass spectrometry (GC-MS) showing minimal changes in key components like citronellol, nerol, and geraniol attributed to stable water use efficiency (WUE). However, lower water and nitrogen levels decreased essential oil yield, indicating a trade-off between stress adaptation and production during flowering. Similarly, Uçar et al. [158] demonstrated that drought (no irrigation) reduced flower production and oil yield in *R. damascena* but enhanced oil quality by increasing citronellol, geraniol, and nerol levels.

We compiled key findings across various rose species and cultivars to provide a comprehensive overview of roses’ morphological, physiological, and biochemical responses to DS. Table 3 summarizes these responses, focusing on the effects of differing DS severities, resistance means, and associated processes. This compilation underscores the complexity of rose adaptation to DS and establishes a foundation for developing targeted strategies to enhance their resilience.

## 4. Influence of Dehydration Stress on Rose Quality and Postharvest Longevity

Water relations are critical in regulating the lifespan of cut flowers, including cut roses, as transpiration-induced water loss significantly shortens vase life and impairs quality during the post-harvest stage [159]. WS occurs when water loss through stomata exceeds absorption, leading to wilting, premature senescence, impaired flower growth and development, and a reduced vase life [124,125,160]. For example, DS in cut roses caused abnormal flower opening and reduced flower size [124,125,161]. Stomatal responses to DS vary across cut rose cultivars; a study on cut roses subjected to long-term dry storage at low temperatures revealed differing stomatal opening and transpiration rates among cultivars [162]. Depending on the cultivar, this variability highlights a strong correlation between stomatal function, DS tolerance, and vase life maintenance [162].

Additionally, exposing cut *R. hybrida* cv. Wild Look to air for 1–3 h without stem submersion in water increased stomatal opening under dark conditions, elevating transpiration rates [163]. Thus, regulating stomatal closure is essential for enhancing DS tolerance and extending vase life in cut roses [164].

DS significantly reduces water availability, impairing the quality and longevity of rose flowers, as evidenced by numerous studies across various DS scenarios and growth stages (Table 4). Beyond its impact on water relations, DS also alters morphological and developmental traits, leading to bud deformation, shortened petal length, and petal deformities in roses, as reported by Shi et al. [42]. These findings align with observations in Damask roses, where DS affects multiple floral traits, including flowering time, flower quantity, size, dry weight, number of petals, receptacle diameter, and individual petal dry weight, confirming its adverse impact on rose quality [41].

## 5. Molecular Responses to Drought Stress

Under adverse conditions, plants have evolved complex adaptive mechanisms to tolerate low soil moisture [166,167]. At the molecular level, plants respond to DS through coordinated regulatory mechanisms involving cellular signalling pathways and transcriptional regulation, enabling specific gene responses to stress [168,169]. Generally, plants react to DS in three key steps: signal perception, signal transduction, and expression of stress responses, which trigger physiological and metabolic adaptations [5,170,171]. Plant cells detect DS stimuli via sensors or receptors primarily on the cell membrane, activating intracellular signaling molecules through second messengers like ROS, Ca^2+^, NO and H_2_O_2_, which then initiate corresponding signaling pathways [172]. Protein phosphorylation and dephosphorylation, mediated by protein kinases and phosphatases, respectively, are critical in these signal transduction pathways, with mitogen-activated protein kinases (MAPKs) and calcium-dependent protein kinases (CDPKs) playing key roles in DS signaling [173]. In the final step, kinases or phosphatases activate or deactivate TFs, which regulate downstream gene expression by binding to specific cis-elements in promoter regions [174]. TFs are further regulated transcriptionally by upstream components [175] and undergo post-transcriptional modifications, such as ubiquitination and sumoylation, for-ming a complex regulatory network that governs stress-responsive gene expression and orchestrates physiological and metabolic processes [176].

**Figure 4:**
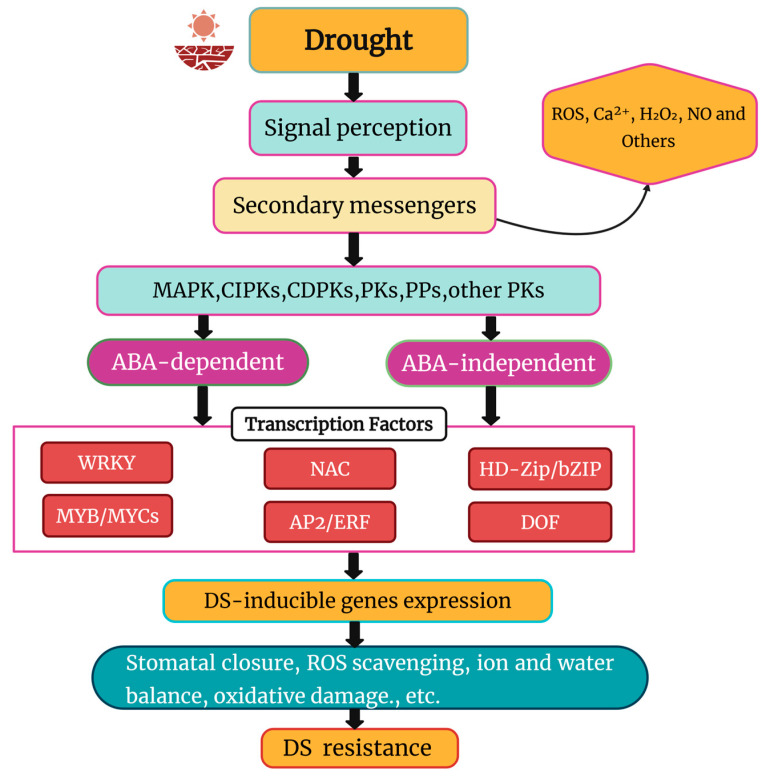
Schematic representation of DS signaling in plants. Modified from Khan et al. [180]. DS triggers ABA-dependent and ABA-independent pathways, activating transcription factors such as MYB/MYCs, WRKYs, HD-Zip/bZIP, NACs, DOF, and AP2/ERF. ROS—Reactive Oxygen Species, Ca^2+^—Calcium ions, H_2_O_2_—Water, NO—Nitric Oxide, MAPK—Mitogen-Activated Protein Kinase, CIPKs—CBL-Interacting Protein Kinases, CDPKs—Calcium-Dependent Protein Kinases, PKs—Protein Kinases, PPs—Protein Phosphatases, ABA—Abscisic Acid.

Figure 4 comprehensively summarizes the genes and signaling pathways involved in DS tolerance.

In plants, DS induces numerous signaling pathways involving many proteins, such as TFs, functional proteins, enzymes, molecular chaperones, and metabolites [177]. Based on genome-wide analysis, a wide range of transcription factor families relevant to DS responses have been identified in different plant species [178]. Plants use a complex system of TFs to control and mitigate drought damage at different stages of plant life during times of low water availability, which is essential for their growth and development [179].

TFs regulate a diverse array of genes across various gene families, including NAC, WRKY, HD-Zip/bZIP, AP2/ERF, and MYB, among others, with their activity influenced by factors such as plant species, cultivar, growth stage, and DS severity [181]. Approximately 7% of coding sequences in plant genomes encode TFs, many of which are involved in the immediate early response to environmental stresses [182]. This section explores the prominent TF families contributing to abiotic stress tolerance in rose species, focusing on their roles in coordinating responses to DS in rose plants.

The NAC family, a prominent group of plant-specific TFs, contains approximately 110 genes in Arabidopsis and 150 genes in rice [183]. NAC TFs have been known for their essential role in plant growth and their ability to modulate plant responses to abiotic stresses, including drought, salt and cold stress [183,184,185,186]. *RcNAC72* is an important TF gene that responds to WD in rose plants [65,187]. Overexpression of *NAC72* in *Arabidopsis thaliana* has been shown to enhance plant resistance to DS, revealing its potential function in regulating ABA-responsive gene expression [187]. In *R. chinensis* cv. Old Blush. *RcNAC72* expression is significantly elevated in response to ABA, water and salt stress, and cold conditions, while its overexpression in *Arabidopsis thaliana* enhances dehydration tolerance; conversely, *RcNAC72* suppression increases DS resistance and reduces water loss in roses [65]. Similarly, *RhNAC2* and *RhNAC3*, two NAC TFs, enhance DS tolerance in rose flowers by regulating genes involved in osmoregulation and cell wall synthesis, respectively [165,188]. In a related mechanism, *RhNAC31* upregulates gene expression to improve tolerance to multiple abiotic stresses in genetically modified plants [189]. Additionally, *RcNAC091* enhances DS response in roses via the ABA-dependent pathways [190]. The NAC protein RD26, expressed under DS conditions, aids plants in regulating ABA-induced gene transcription during abiotic stress [191]. Furthermore, Fu et al. [192] demonstrated that DS activates the RD22 (*RcBURP4*) gene in roses, underscoring its critical role in DS resistance.

The AP2/ERF family of TFs is critical in regulating plant growth, development, and responses to environmental stresses, including drought and salt stress [193,194,195]. In roses, *RcDREB2B*, an AP2/ERF gene, exhibits reduced expression under DS, though plants with higher expression are more sensitive to PEG, ABA, and salinity [45]. A subsequent study indicates that ethylene response factor 109 (*ERF109*) is critical for the DS resilience of roses and significantly contributes to WS regulation [65]. Another AP2/ERF TF, *RcTINY2*, identified in roses, mediates multiple processes and enhances abiotic stress resistance via the ABA pathway [20]. ABA treatment upregulates *RcTINY2* transcription in rose leaves but inhibits it in roots, where NaCl and PEG also suppress its expression; while overexpression of *RcTINY2* increases sensitivity to ABA, NaCl, and PEG in *Arabidopsis*, its suppression in roses has minimal impact on drought or salinity tolerance [65]. These findings highlight the diverse roles of the AP2/ERF TF family in modulating rose responses to DS. Internal signals driving osmotic changes have been extensively studied, revealing that *RhNAP* (involved in ABA responses) and *RhCKX6* (involved in cytokinin degradation) confer DS tolerance in immature flowers of *R. hybrida* cv. Samantha, though this interaction accelerates petal wilting in mature flowers [196]. DS also activates *RhABF2*, a TF in the ABA signaling pathway, which directly induces *RhFer1*, a ferritin gene regulating iron levels during DS; this *RhABF2*/*RhFer1* activation enhances DS resistance and supports flower reopening after drought [196]. Additionally, *P5CS*, encoding pyrroline-5-carbox-ylate synthetase—a key enzyme in proline synthesis—is highly expressed during DS, further aiding stress tolerance [123].

Several studies have confirmed the critical role of Dof TFs in regulating plant responses to abiotic stress, particularly DS [76,197,198]. Studies in maize involving CRISPR/Cas9 and overexpression demonstrated that *ZmDOF22* enhances DS resistance by modulating the ABA pathway, promoting stomatal closure, and reducing water loss [199]. Similarly, *Md-DOF54* in apples positively regulates drought resistance [198]. In kiwifruit, different abiotic stresses induce varying expression levels of six *AcDOF* TFs, with drought-resistant cultivars exhibiting significantly higher *AcDOF22* expression [200]. After analyzing the rose genome, Nan et al. [201] identified 24 Dof genes (from *RchDof1* to *RchDof24*). In response to drought and salinity, most of these genes showed elevated expression levels, indicating the crucial function of Dof genes in the ability of rose plants to tolerate abiotic stress.

MYBs are a core family of TFs regulating secondary metabolism, interacting with hormones and environmental signals, regulating gene expression and cell differentiation, and increasing the plant’s resistance to abiotic stresses such as water deprivation [202]. According to Jia et al. [65], MYB family members and basic helix-loop-helix (bHLH) TFs, especially *bHLH162* and *bHLH35*, are essential for adapting *R. chinensis* to cold and DS. In their research, Shang et al. [203] proposed that *RcMYB8* modulates the action of two proteins, RcPR5/1 and RcP5CS1. RcP5CS1 promotes proline synthesis, enhancing tolerance to osmotic stress, while RcPR5/1 strengthens plant defense mechanisms. Rose plants overexpressing *RcMYB8* showed improved growth, higher survival rates, and enhanced physiological parameters under stress, indicating increased resilience to salinity and DS.

In *R. chinensis* Jacq., researchers studied RcTCP genes during growth stages under DS using a public RNA-seq dataset, finding that without DS, RcTCP genes are significantly increased, highlighting their essential role in protecting against DS [204]. Furthermore, the SOD2 gene, which helps produce superoxide dismutase two in *Escherichia coli*, dramatically improves the rose’s ability to handle DS [205]. On the other hand, reducing the activity of RhHB1—a type of protein that helps control gene activity in rose flowers—raises the levels of jasmonate-isoleucine (JA-Ile) and lowers the plant’s ability to tolerate DS; RhHB1 attaches to the *RhLOX4* gene’s control region, inhabiting the production of lipoxygenase 4 (*RhLOX4*) [206].

Mediator’s multiprotein complex acts as an intermediary between RNA polymerase II (Pol II) and DNA-binding transcription factors. It is essential for transcription initiation, elongation, and splicing, among other steps of transcription [207,208]. Moreover, plants’ ability to adapt to various abiotic stresses, including dehydration, cold, and salinity, depends on the mediator modules [209,210]. Recent studies have investigated the function of some mediators in DS tolerance. DS rapidly activates *RhMED15a*, a mediator in roses, which ABA and MeJA modulate. Inhibition of *RhMED15a* led to a significant decline in DS tolerance [203].

Furthermore, the scientists identified a novel mediator, *RhMED15a-like,* in hybrid rose plants. WS and ABA/MeJA treatment decreased its expression. Silencing *RhMED15a-like* in roses decreased DS tolerance while overexpressing it in Arabidopsis increased osmotic stress tolerance. Moreover, genes of the JA and ABA signalling pathways were more expressed when *RhMED15a-like* was silenced, while genes that interact with stress were less expressed. Indicating that *RhMED15a-like* enhances the drought resistance of roses by regulating genes that interact with stress hormones and drought signaling pathways [105]. A genome scan of *R. chinensis* identified 41 genes encoding CCHC-type zinc finger proteins (RcCCHC-ZFPs), with *RcCCHC25* showing significant overexpression in response to lack of water conditions [211]. The qRT-PCR test showed that *RcHSP90-1*, *RcHSP90-5*, and *RcHSP90-6* are crucial for controlling how cells react to water and salt stress. The findings show that CCHC-type zinc finger proteins and HSP90 genes play a part in *R. chinensis*’s ability to survive under WS [212].

A study on the WRKY gene family in roses identified eight *RcWRKY* genes that respond to heat, salinity, and DS conditions, exhibiting both positive and negative regulatory effects on plant stress responses; the rapid activation of *RcWRKY14* and *RcWRKY16* highlights their potential as candidate genes for further exploring stress resistance pathways in roses [213]. DS significantly impacts cut rose lifespan, particularly in ethylene-sensitive varieties, by increasing ethylene production through the upregulation of biosynthetic genes *RhACS1* and *RhACS2* (encoding ethylene-1-aminocyclopropane-1-carboxylate synthase), especially in sepals, and by modifying the ethylene receptor gene *RhETR3* [161]. Ethylene-sensitive roses under DS sustainably produce ethylene, which impairs flower development and reduces vase life, as reported by Sukpitak et al. and Dar et al. [164,214]. In rose petals, five superoxide dismutase (SOD) genes were analyzed, with four—*RhMnSOD1*, *RhCu*/*ZnSOD2*, *RhCu*/*ZnSOD1*, and *RhCu*/*ZnSOD3*—showing significant functional decline after cell dehydration, though their expression levels normalized post-rehydration [125]. In Arabidopsis, DS activates MAPK cascade components like *MPK4 and MPK6*, underscoring their role in DS signaling [215,216,217]. Similarly, *RhMKK9*, a MAPK cascade component in cut roses, exhibits rapid, high expression in the gynoecium upon desiccation; silencing *RhMKK9* significantly reduces ethylene production during rehydration, thereby affecting flower growth and longevity [218]. Indicating that the MAPK cascade is critically essential in plant responses to DS and rehydration, emphasizing its importance in controlling responses to water availability.

Generally, studying TFs and how they contribute to WS tolerance in roses has important implications for improving drought resistance in this valuable ornamental crop.

## 6. Different Strategies to Mitigate the Adverse Effects of Drought Stress on Rose Plants

**Figure 5:**
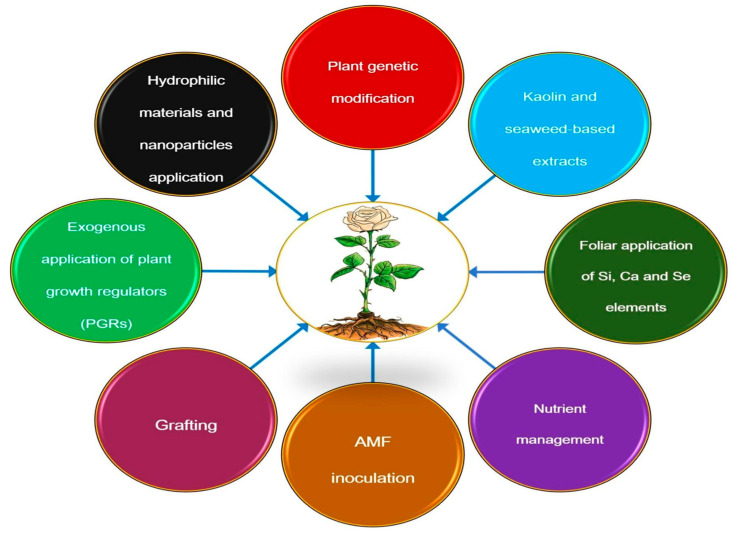
Schematic representation of an integrated management strategy to improve plant resistance to DS.

To mitigate the adverse effects of DS on plants, effective strategies such as genetic control, soil management, water delivery, foliar spraying, and mulching should be implemented [219], as illustrated in Figure 5. These strategies are discussed in detail below.

Arbuscular mycorrhizal fungi (AMF) are common soil microorganisms that form symbiotic relationships with the roots of approximately 90% of plant species, including angiosperms [220,221]. This symbiosis improves soil structure and enhances plant resist-ance to DS through various physiological, physical, nutritional, and cellular processes [38,222,223,224]. AMF supports seedling survival, facilitates water absorption and transport, modifies root architecture, enhances the effects of plant-produced hormones, and accelerates the removal of ROS from plant cells [108,225,226,227,228].

Multiple studies demonstrate that AMF mitigates DS in rose crops. For instance, AMF-treated Damask roses exhibited reduced susceptibility to DS due to enhanced physiological, biochemical, and water retention factors, leading to increased flower production [38]. Similarly, Augé et al. [229] reported that *R. hybrida* cv. Love, colonized by *Glomus intraradices* Schenk and Smith showed greater DS resistance attributed to elevated levels of free amino acids and carbohydrates in the roots. Additionally, *Glomus intraradical* significantly improved photosynthesis in *R. hybrida* cv. New Dawn under DS conditions [230]. Inoculation with either *Glomus deserticola* Trappe, Bloss, and Menge or *Glomus intraradices* Schenk and Smith also alleviated DS effects in *R. hybrida* cv. Samantha [231,232]. Green et al. [233] observed that leaves of *Glomus intraradices*-treated roses exhibited reduced transpiration, suggesting that harvested flowering stems may have an extended vase life.

Plant growth regulators (PGRs), including ABA, salicylic acid (SA), and JA, as well as cytokinins, are effective in promoting plant growth and productivity under water limitation conditions [228,234]. ABA enhances DS resistance by reducing transpiration and sustaining photosynthesis [97,235,236]; its application in spring or summer further improves water retention in *R. hybrida* flowers, extending their lifespan [237].

Applying nanoparticles offers a novel approach to alleviate DS, as they induce morphological, biochemical, and physiological changes in plants, enhancing DS tolerance by increasing hydraulic conductance, improving root water uptake, modulating proteins involved in hormone pathways, stress signa, redox processes, and ROS scavenging [238,239]. Foliar application of metal oxide nanoparticles, such as titanium dioxide (TiO_2_), iron oxide (Fe_3_O_4_), and zinc oxide (ZnO), has been shown to improve metabolic and physiological processes in plants under DS [240]. Several studies, such as those by Siddiqui et al. and Seleiman et al. [32,241], have shown that silicon nanoparticles (Si-NPs) can help plants cope with the adverse effects of abiotic stresses such as water and salt. Research conducted by Seyed Hajizadeh et al. and Alavi et al. [39,81] clarified that Si-NPs and TiO_2_ nanoparticle applications enhanced the growth traits, WUE, and mineral uptake of rose plants during WS.

Exogenous application of nutrients and chemicals offers an effective strategy to mitigate the adverse effects of DS on plants. Calcium (Ca^2+^), a critical nutrient in plant responses to environmental stresses like DS, reduces lipid peroxidation and enhances antioxidant enzyme activity, thereby alleviating DS-induced damage [242,243]. For example, Zhao et al. [64] demonstrated that exogenous Ca^2+^ application significantly improved DS resistance in roses. Similarly, silicon (Si) helps plants withstand abiotic stresses, including heavy metals, salinity, and DS, by supporting growth and survival [244,245,246,247]. Foliar silicon and potassium silicate application on Damask rose seedlings, under both optimal and DS conditions, enhances flower growth and increases essential oil production [46,51]. These findings suggest that exogenous Ca^2+^ and Si applications effectively mitigate DS impacts on roses and other crops. Additionally, polyamine treatment alleviates DS-induced stress, with spermine (Spm) or spermidine (Spd) at 0.5 mmol, improving plant growth and physiological traits in Damask roses under DS [248]. Moftah et al. [249] also revealed that kaolin application to the ornamental plant *Polianthes tuberosa* L. improves its water status, water use efficiency (WUE), and photosynthetic activity under DS conditions.

**Figure 6:**
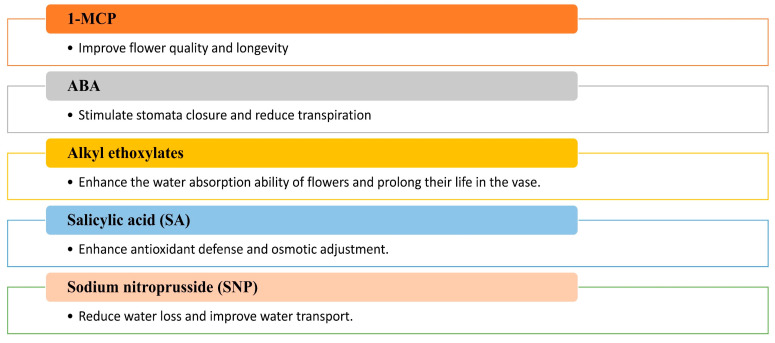
Effects of exogenous application of synthetic compounds (1-MCP, alkyl ethoxylate, SNP) and phytohormones (ABA, SA) on the drought tolerance of rose flowers.

In line with these findings, Sukpitak et al. [164] noted that floricultural products can be assisted in coping with WS through the exogenous application of chemical treatments, including ethylene inhibitors, select phytohormones, and other substances, as shown in Figure 6.

Pre-treatment with ethylene inhibitors before DS can mitigate its adverse effects on cut flower longevity, as observed by Sukpitak et al. [164]. For instance, 1-methylcyclopropene (1-MCP), an ethylene action inhibitor, can be applied to cut flowers of *R. hybrida* cv. Samantha, before dehydration, prevents DS-induced impairments in flower opening and petal cell growth [161]. Similarly, ABA minimizes water loss in flowers by rapidly inducing stomatal closure in chrysanthemum petals during DS, thereby reducing transpiration [162,164]. ABA treatment also maintains the marketable quality of flowers post-rehydration under harsh conditions and, in the Akito cultivar of cut roses, soaking in 0.1 mM ABA enhances stomatal closure, further reducing transpiration [162]. Based on these investigations, using ABA can improve the quality and longevity of post-harvest floral products and water consumption efficiency.

Apart from ABA, antitranspirant compounds like sodium nitroprusside (SNP), a nitric oxide (NO) donor [250], and acetylsalicylic acid [251] decrease water loss from leaf tissue by reducing the size and number of stomata, thus promoting a balanced water supply [252]. These compounds are expected to extend the vase life of partially dehydrated cut flowers [253]. A case in point is that the cut flowers of three rose cultivars, Testarossa, Bordeaux, and Lenny, were placed in vase water containing 15 mM SNP (an NO inducer) or 15 mM acetylsalicylic acid (known by Aspirin) under mild desiccation (12% weight loss). The data showed that treatments that slow the transpiration rate by closing the stomata can significantly reduce the adverse effect of mild desiccation on vase life. This study was conducted by Fanourakis et al. [253]. In addition, conditions that facilitate water absorption, such as adding surfactants to the vase water [254,255], can reduce the adverse effects of partial dehydration on vase life. Van Doorn et al. [255] reported that certain alkyl ethoxylate mixtures can significantly improve the ability of cut roses and Bouvadia flowers to uptake water and last longer in the vase.

Mulching, an effective agronomic practice to mitigate DS, enhances soil moisture retention, regulates soil temperature, and improves fertility [256]. Horo et al. [257], mulching suppresses weed growth and increases moisture retention, improving agronomic and floral characteristics in rose plants. Comparable findings were reported by Sardar et al. [258] in *R. centifolia* and by Tripathy et al. [234] in rose cv. ‘Mainu Parle’, who demonstrated that black polythene mulch (300 micron) significantly increased flowering duration and flower number, supporting its role in DS mitigation by potentially in alleviating water retention and reducing different physiological stress indicators.

## 7. Conclusions and Future Perspectives

DS continues to pose a significant challenge to the global ornamental and cut flower industries, with rose production particularly affected due to declines in ornamental quality and economic value. This review has demonstrated that DS triggers a wide range of morphological, physiological, biochemical, and molecular responses in rose plants. A comprehensive understanding of these mechanisms is essential to formulate innovative approaches to mitigate the adverse effects of water shortages. Current strategies focus on developing drought-resistant varieties, genetic modification, and implementing natural drought-tolerant genotypes. Additionally, emerging treatments involving plant hormones such as ABA and GA, combined with nanomaterials like TiO_2_and Si-NPs, offer promising prospects for enhancing drought resistance. However, significant research gaps remain, especially in the genetic regulation of drought responses in roses, which may reveal valuable genetic stress memory mechanisms for breeding programs.

Furthermore, the effect of drought on the biosynthesis of secondary metabolites, including essential oil synthesis, requires further study to improve yield and quality under water-limited conditions. Root system architecture’s role in improving water absorption efficiency remains insufficiently studied. Expanding the study to include multiple commercial and wild rose cultivars may reveal distinct adaptive characteristics while examining the interactions of various stresses (e.g., drought with heat or salinity), which will improve breeding approaches based on cross-tolerance. Future research should focus on identifying genetic and transcriptional networks associated with drought adaptation, enhancing precision irrigation and soil moisture management tailored to rose cultivation, and evaluating the synergistic effects of hormone and nanotechnology applications under field conditions. Furthermore, incorporating genomic selection and gene editing techniques, such as CRISPR, into breeding programs may accelerate the development of drought-resistant rose varieties. Addressing these deficiencies is crucial to sustaining rose production under increasing water scarcity problems, thus ensuring this critical horticultural crop’s environmental sustainability and economic viability.

## Figures and Tables

**Table 1:** Hormonal Changes in Rose Plants in Response to DS.

Cultivar	Hormonal Changes	Key Findings	References
*R. rubiginosa* (Sweet briar)	ABA ↑ (3-fold increase).	Prolonged DS contributes to the accumulation of ABA, which is essential in the stress response.	[106]
	GA3, GA4, GA5, GA6 ↑ (329.8% increase). GA7, GA8, GA9 ↓ (65.5% decrease). Kinetin riboside ↑ (136.2% increase).	High concentrations of kinetin riboside may be associated with reduced growth and increased tolerance. Decreased levels of some gibberellins may enhance the ability of rose plants to withstand DS by inhibiting growth and reallocating energy toward defense mechanisms.	[107]
*R. chinensis* Jacq. var. minima Rehd.	↓ IAA, ↓ Zeatin, ↓ GA (with six-day irrigation intervals and a salinity of 4 dS/m). ↑ ABA (With 4 dS/m salinity).	Salinity and extreme WS reduce IAA and zeatin content. Severe water deficits and salinity stress also reduce GA in combination with longer irrigation intervals. ABA levels increase with higher salinity levels.	[107]
*R. chinensis* Jacq.	↑ ABA (leaves and roots), ↓ ICA (leaves), ↑ ICA (roots), ↓ IAA (leaves), ↑ IAA (roots, MD), ↓ IAA (roots, SD), ↑ ME-IAA (leaves and roots), ↓ IP (leaves), ↑ IP (roots).	DS induces complex hormonal changes in leaves and roots, with ABA playing a greater role in dehydration responses, particularly in leaves.	[45]
*R. hybrida* cv. Samantha	↑ JA-Ile (petals).	Increased JA-Ile in petals impairs osmotic adjustment, reducing resistance to water stress.	[108]

↑—Increase; ↓—Decrease; DS—Drought Stress; MD—Mild Drought; ABA—Abscisic Acid; GA—Gibberellin; IAA—Indole-3-acetic Acid; ICA—Indole-3-carboxaldehyde; ME-IAA—Methylindole-3-acetic Acid; IP—Indole-3-propionic Acid; JA—Jasmonic Acid.

**Table 2:** Antioxidant enzyme activity in response to DS.

Genotype	DS Conditions	Enzymes	Activity	References
*R. hybrida* cv. Samantha.	Rose flowers dehydration	SOD and APX	Elevated by WS	[124]
R. laevigata	Light and DS	SOD and POD	(+) of both enzymes under light DS. (−) under severe DS.	[134]
*R. hybrida* cv. Samantha.	Rose flower dehydration	SOD and Cu/ZnSOD	(+) SOD and Cu/ZnSOD in dehydration. (−) SOD and Cu/ZnSOD after rehydration.	[125]
*R. chinensis* cv. Old Blush	Gradual drought over (30, 60, 90) days	SOD	(+) SOD activity with increasing DS duration. Maximum SOD activity reached after 90 days of DS treatment. (−) SOD content after re-watering.	[65]
*R. damascena*	100% FC (control), 50% FC (moderate stress), 25% FC (severe stress)	AChE and LOX. inhibitory enzyme activities	Increased antioxidant and inhibitory enzyme activities in both mild and extreme water shortage conditions.	[37]
*R. damascene*; *R. canina*	(25%, 50%, 100% FC)	CAT POD SOD	(+) of all three enzymes *R. damascena* showed higher SOD and CAT activity compared to *R. canina*. No significant difference in POD activity between the two species.	[75]
*R. bracteata.*, *R*. *chinensis*., *R. rouletii*, *R. foetida*; *R. indica L*., *R. gallica*., *R. hugonis* and cv. Borisphen	Controlled leaf wilting under varying temperatures and humidity.	CAT, POD, PPO	(+) of all three enzymes under wilting conditions. *R. rouletii*, *R. foetida*, and *R. gallica* showed sustained enzyme activity even after recovery, suggesting more severe metabolic disruption.	[135]
*R. damascena*	Different PEG concentrations	TAA	(+) (TAA)	[39]
*R. hybrida*	FI (full irrigation), SDI (moderate DS), PRD1 (severe DS), PRD2 (partial root DS)	CAT and APX	Less activity of APX and CAT in control conditions (FI). Upregulation of these enzymes in response to WS.	[81]
*R. damascena* cv. Kashan 93	Three distinct WD levels (70, 40, and 10% of available water content).	CAT, POD and APX	The three antioxidant enzymes were activated by a lack of water.	[41]

(+)—Increase; (−)—Decrease; SOD—Superoxide dismutase; APX—Ascorbate peroxidase; Cu/ZnSOD—Copper-zinc-superoxide dismutase; POD—Peroxidase; AChE—Acetylcholinesterase; LOX—lipoxygenase; CAT—Catalase; POD—Peroxidase; PPO—Polyphenol oxidase; TAA—Total antioxidant activity.

**Table 3:** Morphological, physiological, and biochemical responses of rose species and cultivars to DS: Effects of severity and resistance mechanisms.

Rose Variety	Severity of DS	DS Resistance	Key Findings	Reference
*R. damascena* Mill.	Mild (50% FC), Severe (25% FC)	Medium	Growth reduced (fresh/dry weight down); Pn and Gs increased 31% and 19% (mild), decreased 55% and 36% (severe); RWC and LWP decreased (72% in severe); chlorophyll b decreased 54% (severe); proline increased 34.6% (severe); soluble sugars up 33.6%.	[40]
*R. damascena* cv. Maragheh	Severe (PEG-induced)	Low	Chlorophyll decreased 30%; K^+^ and P decreased 56% and 52%; leaf number and area reduced 40%; TAA increased.	[39]
*R. damascena* cv. Kashan	Severe (PEG-induced)	Low	Chlorophyll decreased 41%; K^+^ and P decreased 47% and 52%; TAA increased.	[39]
*R. damascena* var. trigintipetala	Mild (50% FC), Severe (25% FC)	Medium	Growth reduced; proline increased 34.6% (severe); soluble sugars up 33.6%; SOD and CAT increased; K^+^ increased; other nutrients decreased.	[37]
*R. hybrida* cv. Charming Black	Severe DS	Low	Shoot length, weight, and leaf area reduced; Pn decreased; fresh weight lower than control; photosynthetic pigments decreased.	[42]
*R. hybrida* cv. Club Nika	Mild (75% irrigation), Moderate (50% irrigation).	Medium	Fresh/dry stem weight decreased 19% (mild) and 36% (moderate); bud sprouting was delayed; no change in photo-synthetic pigments.	[44]
*R*. *chinensis* cv. Old Blush	Severe (90 days without water)	Medium	Leaves wrinkled after 30 days; severe wilting at 90 days; full recovery after rehydration; SOD peaked at 90 days, decreased after rewatering; MDA peaked at 90 days; lowest LWC and SWC at 90 days.	[65]
*R. fortuniana*	Cyclic DS	High	Superior vegetative growth and leaf area; highest drought tolerance among rootstocks.	[59]
*R. odorata*		Low	Severe reduction in growth; lowest drought tolerance among rootstocks.	
*R. multiflora*		Medium	Intermediate growth reduction; moderate tolerance.	
*R. hybrida* cv. Dr. Huey		Medium	Intermediate growth reduction; moderate tolerance.	
*R. rugosa*	Severe (PEG 5%)	High	Pn and Gs improved; Tr decreased 29.42%; structural adaptations (sunken stomata, low stomatal density); increased MDA and oxygen with DS severity.	[67]
*R. canina*	Mild (50% FC), Severe (25% FC)	Medium	Higher chlorophyll under DS; increased H_2_O_2_ and MDA; SOD and CAT increased; proline increased (14.5 mM to 75.5 mM at 25% FC).	[123]
*R. hybrida* cv. Samantha	Severe (dehydration)	Medium	SOD and APX elevated; Cu/ZnSOD increased during dehydration, decreased after rehydration; JA-Ile increased in petals; H_2_O_2_ and MDA increased, decreased upon rehydration.	[124,125]
*R. laevigata*	Light (mild), Severe	Medium	SOD and POD increased under mild DS, decreased under severe DS.	[134]
*R. rubiginosa*	(drought in dry sites)	Medium	ABA increased 3-fold; higher soluble carbohydrates in dry sites; slower water loss; decreased photosynthetic activity.	[106]
*R. chinensis* Jacq. var. minima Rehd.	Severe (6-day irrigation intervals + salinity)	Low	IAA, zeatin, and GA decreased; ABA increased with higher salinity and severe growth reduction.	[107]
*R. roxburghii* (Gui 2 and Gui 7)	DS	Medium	Nitrogen and P decreased under DS; no change in K^+^; proline and soluble sugars increased.	[93]

FC—Field capacity; PEG—Polyethylene glycol; DS—Drought Stress; Pn—Net photosynthetic rate; Gs—Stomatal conductance; RWC—Relative water content; LWP—Leaf water potential; K^+^—Potassium ion; P—Phosphorus; TAA—Total antioxidant activity; SOD—Superoxide dismutase; CAT—Catalase; MDA—Malondialdehyde; LWC—Leaf water Content; SWC—Soil water content; Tr—Transpiration rate; H_2_O_2_—Hydrogen Peroxide; APX—Ascorbate peroxidase; Cu/ZnSOD—Copper/Zinc superoxide dismutase; JA-Ile, Jasmonoyl-Isoleucine; POD—Peroxidase; ABA, Abscisic Acid; IAA, Indole-3-acetic acid; GA—Gibberellic acid.

**Table 4:** Summarize the impact of WS on water relations, rose flower quality, and longevity.

Flowers	Dehydration Treatment	Harvest Stage	Water Relations	Flowers Longevity	References
*R. hybrida* cv. Wild look	Exposure to air for 3 h	Commercial stage	Enhanced transpiration in dark conditions reduced water uptake.	Accelerated bent neck and stimulated petal abscission. Decreased flower quality.	[163]
*R. hybrida* cv. Samantha	Dehydration for 60 h	Completely opened bud	Increased fresh weight loss	29% shorter vase life. Irregular flower opening and loss of market quality.	[125]
	Air-drying for 24 h	Completely opened bud	The water potential declined from −0.5 MPa to −3.4 MPa. The flowers experienced a significant reduction in weight, losing 25.3% of their freshness within 24 h.	Flowers wilting and neck bending. The flowers are vertically compressed and have a low flower height-to-diameter ratio.	[159]
	Dehydration for 24 h	Completely opened bud	Flowers’ initial weight was reduced by 22.8%. The water potential dropped from −0.5 MPa to −3.2 MPa.	Petal cell expansion was inhibited. Abnormal flower openings and shapes.	[161]
	Cyclic dehydration in the air for 48 h	Completely opened bud	Variations in fresh weight were reflected in variations in water potential. The fresh weight experienced a linear decrease, resulting in a loss of approximately 20%. During the first hour of rehydration, flowers abs-orb water very quickly, returning to their initial fresh weight in about 3 h.	Flower stems are bent, and petals are noticeably withered. Petal expansion inhibition. Abnormal flower opening. Decreased market quality	[165]
